# Interactions between Two Different G Protein-Coupled Receptors in Reproductive Hormone-Producing Cells: The Role of PACAP and Its Receptor PAC1R

**DOI:** 10.3390/ijms17101635

**Published:** 2016-09-26

**Authors:** Haruhiko Kanasaki, Aki Oride, Tomomi Hara, Tselmeg Mijiddorj, Unurjargal Sukhbaatar, Satoru Kyo

**Affiliations:** Department of Obstetrics and Gynecology, School of Medicine, Shimane University, 89-1 Enya-cho, Izumo, Shimane 693-8501, Japan; oride@med.shimane-u.ac.jp (A.O.); t-hara@med.shimane-u.ac.jp (T.H.); tselmeg@med.shimane-u.ac.jp (T.M.); unur@med.shimane-u.ac.jp (U.S.); kyo@med.shimane-u.ac.jp (S.K.)

**Keywords:** kisspeptin, GnRH, TRH, PACAP

## Abstract

Gonadotropin-releasing hormone (GnRH) and gonadotropins are indispensable hormones for maintaining female reproductive functions. In a similar manner to other endocrine hormones, GnRH and gonadotropins are controlled by their principle regulators. Although it has been previously established that GnRH regulates the synthesis and secretion of luteinizing hormone (LH) and follicle-stimulating hormone (FSH)—both gonadotropins—from pituitary gonadotrophs, it has recently become clear that hypothalamic GnRH is under the control of hypothalamic kisspeptin. Prolactin, which is also known as luteotropic hormone and is released from pituitary lactotrophs, stimulates milk production in mammals. Prolactin is also regulated by hypothalamic factors, and it is thought that prolactin synthesis and release are principally under inhibitory control by dopamine through the dopamine D2 receptor. In addition, although it remains unknown whether it is a physiological regulator, thyrotropin-releasing hormone (TRH) is a strong secretagogue for prolactin. Thus, GnRH, LH and FSH, and prolactin are mainly regulated by hypothalamic kisspeptin, GnRH, and TRH, respectively. However, the synthesis and release of these hormones is also modulated by other neuropeptides in the hypothalamus. Pituitary adenylate cyclase-activating polypeptide (PACAP) is a hypothalamic peptide that was first isolated from sheep hypothalamic extracts based on its ability to stimulate cAMP production in anterior pituitary cells. PACAP acts on GnRH neurons and pituitary gonadotrophs and lactotrophs, resulting in the modulation of their hormone producing/secreting functions. Furthermore, the presence of the PACAP type 1 receptor (PAC1R) has been demonstrated in these cells. We have examined how PACAP and PAC1R affect GnRH- and pituitary hormone-secreting cells and interact with their principle regulators. In this review, we describe our understanding of the role of PACAP and PAC1R in the regulation of GnRH neurons, gonadotrophs, and lactotrophs, which are regulated mainly by kisspeptin, GnRH, and TRH, respectively.

## 1. Introduction

Female reproductive functions are elaborately controlled by the hypothalamic-pituitary-gonadal (HPG) axis, which mediates input trafficking between the brain and gonads. For many years, gonadotropin-releasing hormone (GnRH) has been thought to be at the highest level in the HPG axis. In short, GnRH is released into hypophyseal portal circulation from GnRH neurons in the hypothalamus and then reaches the anterior pituitary gland. GnRH binds to the GnRH receptor (GnRHR) within the gonadotrophs and stimulates the release and synthesis of the gonadotropins luteinizing hormone (LH) and follicle-stimulating hormone (FSH) [1]. Most recently, after the discovery of inactivating mutations in the kisspeptin receptor (Kiss1R) in patients with hypogonadotropic hypogonadism [2,3], it was revealed that hypothalamic kisspeptin is a stimulator of GnRH and is positioned upstream of GnRH in the HPG axis. Now, it is generally agreed that kisspeptin-secreting neurons within the hypothalamus activate GnRH neurons through Kiss1R. Thus, GnRH stimulates the synthesis and release of LH and FSH, which eventually regulate sex steroid synthesis or gametogenesis in the gonads [4,5,6]. In addition to gonadotropins, prolactin is another important pituitary hormone with diverse actions, including a role in reproduction [7]. As with the HPG axis, neuroendocrine regulation of prolactin was previously established, and it is generally agreed that the hypothalamus predominantly exerts an inhibitory influence on lactotrophs through dopamine [8]. However, prolactin-releasing factors exist as well, the principal one being thyrotropin-releasing hormone (TRH).

The hypothalamic factors kisspeptin, GnRH, and TRH are the principle regulators for the control of GnRH, gonadotropins (LH and FSH), and prolactin, respectively. However, it is evident that hypothalamic peptides other than kisspeptin, GnRH, and TRH could participate in the control or modification of hormone-secreting cells. Pituitary adenylate cyclase-activating polypeptide (PACAP), a known multifunctional peptide from the hypothalamus, is one of the candidates to modify the secretory response of neuroendocrine cells. There are many articles in last a few years regarding PACAP, and we have also previously published a review article that described the role of PACAP in the cell models of the pituitary gonadotroph and GnRH-producing neuron [9]. In this review, we further focused on the role of PACAP in modulating neuroendocrine cell functions in GnRH-, gonadotropin-, and prolactin-producing cells. Interactions between PACAP and their principle regulator, kisspeptin, GnRH, and TRH, are discussed based on our observations using cell models.

## 2. Pituitary Adenylate Cyclase-Activating Polypeptide (PACAP) and Its Receptor

Over 20 years ago, Arimura and colleagues isolated a novel neuropeptide from the sheep hypothalamus based on its ability to stimulate cAMP production in anterior pituitary cells [10]. From sequence identity, PACAP was found to be a highly conserved member of the secretin/glucagon/vasoactive intestinal peptide superfamily [11]. PACAP can exist in either a 38-amino acid (PACAP38) or 27-amino acid (PACAP27) form, each derived from the same precursor protein encoded by the *Adcyap1* gene. In the hypothalamus, PACAP expression is localized to the cell bodies in the hypothalamic paraventricular, supraoptic, and arcuate nuclei, with a neuronal fiber network oriented towards the median eminence and pituitary stalk, which abut hypophyseal portal capillaries [12,13,14]. These observations suggest that PACAP probably acts within the hypothalamus and on pituitary cells as a hypophysiotropic hormone. PACAP binds to three receptor subtypes that are all coupled with G protein: the PACAP type 1 receptor (PAC1R), the vasoactive intestinal polypeptide 1 receptor (VPAC1R), and VPAC2R. PACAP receptors are expressed in the central nervous system as well as peripheral organs [15]. PACAP receptors have been detected in the preoptic area (POA) in the hypothalamus where GnRH neurons are found [16], and in the anterior pituitary where gonadotrophs and lactotrophs reside [17].

## 3. Hypothalamic Kisspeptin Controls GnRH Neurons

Although hypothalamic GnRH has been defined as the final neuronal component of the HPG axis, kisspeptin has been identified as playing an essential role in the regulation of GnRH. In short, it is generally currently agreed that kisspeptin is positioned upstream of GnRH. Kisspeptin fibres project to GnRH neuronal cell bodies and processes, and GnRH neurons possess Kiss1R [18]. Kisspeptin causes depolarization of GnRH neurons and increases their firing [19,20]. Indeed, it was reported that kisspeptin stimulates the secretion of GnRH in hypothalamic explants [21] and GnRH mRNA expression within the cell bodies of GnRH neurons [22].

In mammals, kisspeptin neurons have been shown to be located in two different brain regions, both of which are afferent to GnRH neurons. The most prominent population is located in the arcuate nucleus (ARC), where kisspeptin is coexpressed with dynorphin and neurokinin B. Kisspeptin neurons within the ARC are responsible for the GnRH pulse generator. Another kisspeptin population has more variable neuroanatomical distribution, depending on the species. In rodents, it is localized in the anteroventral periventricular nucleus (AVPV) of the hypothalamus, where kisspeptin neurons are known to be linked to the timing of the pre-ovulatory GnRH/LH surge in females. In primates, this population is mainly located within the POA [4,23]. Taken together, the results suggest that kisspeptin released from these two populations stimulates GnRH neurons in the hypothalamus to release GnRH into the hypothalamic-pituitary portal circulation, resulting in the release of gonadotropins from the anterior pituitary [24]. 

### 3.1. Kiss1R and PACAP within GnRH Neurons

Kiss1R couples with the protein Gq/11 to induce intracellular calcium mobilization [25,26]. Kisspeptin signaling was further confirmed by the observation that Kiss1R stimulation induces the formation of inositol triphosphate (IP3) through phospholipase C (PLC)-dependent mechanisms [25,27]. Kisspeptin is a strong activator of extracellular signal-regulated kinase (ERK) in GnRH-producing GT1-7 cells, and cAMP/protein kinase A (PKA)-mediated pathways are also activated by kisspeptin in these cells [28]. ERK, p38 mitogen-activated protein (MAP) kinase, as well as PI3/Akt activation are also stimulated by kisspeptin [25,26,29].

PAC1R is expressed in GnRH-producing GT1-7 cells. PAC1R couples with both Gs and Gq proteins, which accelerates the adenylate cyclase and PLC signaling pathways. The former increases the accumulation of cAMP and activates PKA, whereas the latter activates PKC, which leads to the activation of ERK [10].

### 3.2. Kisspeptin and PACAP Cooperate in GnRH-Producing Neurons

**Figure 1:**
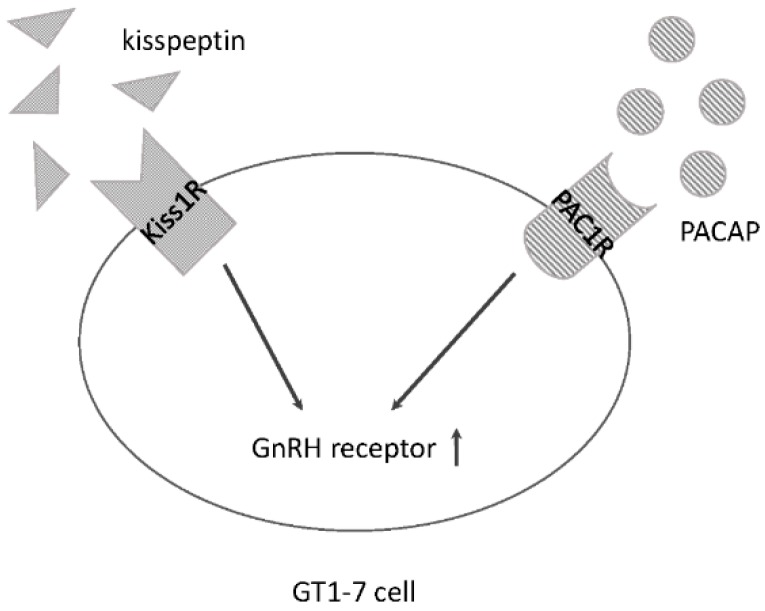
Schematic summary of action of kisspeptin and PACAP in GnRH-producing GT1-7 cells. GT1-7 cells possess Kiss1R and PAC1R, and respond to kisspeptin and PACAP. GnRH receptor expression in GT1-7 cells is increased by these peptides.

A number of studies have demonstrated that GnRH-producing GT1-7 cells respond to kisspeptin and increase their synthesis and secretion of GnRH [22,30]. In contrast, our line of GT1-7 cells did not respond to kisspeptin and failed to increase GnRH expression. However, we found that kisspeptin could increase the expression of GnRHR in GT1-7 cells [28]. PACAP may modulate the HPG axis by acting on GnRH neurons. It was reported that intraventricular injection of PACAP or injection into the medial basal hypothalamus, which contains GnRH neurons, supressed GnRH pulsatility in ovariectomized ewes [31]. Neonatal PACAP administration in rats delays puberty, with decreasing GnRH immunostaining in the hypothalamus [32]. By contrast, it was reported that intracerebroventricular injection of PACAP led to a slight increase in GnRH gene expression in male rats [33]. In this context, the direct action of PACAP on GnRH-producing cells is still contradictory. In experiments using GT1-7 cells, we found that PACAP increased GnRHR expression, but not that of GnRH [34]. Combined stimulation of GT1-7 cells with kisspeptin and PACAP led to higher GnRHR expression levels than the levels achievable with individual treatment, with a concomitant increase in cAMP/PKA pathway stimulation [34] (Figure 1). Although it remains obscure precisely how PACAP affects GnRH neurons, it is plausible that it modulates reproductive function by modulating GnRH neuronal functions.

## 4. Gonadotropin Secretion from Pituitary Gonadotrophs

The gonadotropins LH and FSH are regulated by GnRH, and synthesis and release of LH and FSH from pituitary gonadotrophs is specifically under the control of GnRH pulse frequency. GnRH neurons exhibit a pulsatile pattern of coordinated, repetitive GnRH release into the hypophyseal portal circulation, and gonadotrophs are exposed to pulsatile GnRH. Changes in pulse frequency determine the predominant synthesis and release of gonadotropins. That is, a higher frequency of GnRH increases LH, whereas a lower frequency of GnRH decreases LH but increases FSH [35]. The pattern of GnRH pulses varies physiologically during the reproductive cycle [6]. 

### 4.1. GnRHR Signalling in Pituitary Gonadotrophs

GnRHR is a member of the seven-transmembrane G protein-coupled receptor family. The initial phase of GnRH action involves Gq-mediated PLC stimulation, which leads to the formation of IP3 and diacylglycerol (DAG). IP3 induces the elevation of intracellular calcium and DAG activates PKC, which ultimately activates ERK [36,37,38]. GnRHR also couples with Gs protein to increase cAMP accumulation in LbT2 cells [39]. It is obvious that ERK activation is important in GnRH-induced a-, LHb-, and FSHb-subunit expression [40,41,42]. We have previously examined the activation of ERK by different GnRH pulse stimulation using perifused gonadotrophs. After a GnRH pulse, ERK phosphorylation was rapidly increased, but was not sustained and returned to baseline levels. A similar pattern of ERK activation was observed following a subsequent GnRH pulse. Interestingly, the patterns of ERK phosphorylation in response to pulsatile GnRH at high and low frequencies were distinct, and the duration of ERK phosphorylation in low-frequency GnRH pulses was longer than that induced by high-frequency GnRH pulses [43]. Changes in the pattern of ERK activation by different modes of GnRH pulses might be due to distinct patterns of MAP kinase phosphatase (MKP) expression. MAPs are a family of protein phosphatases that inactivate ERK by dephosphorylation of threonine and/or tyrosine residues [44]. MKP expression was predominantly increased following high-rather than low-frequency GnRH pulses [45]. Because MKP induced by high-frequency GnRH pulses dephosphorylates ERK, which is activated by the initial GnRH pulse, ERK phosphorylation induced by a single GnRH pulse during high-frequency GnRH pulse stimulation would return to the basal level more rapidly.

### 4.2. Effect of PACAP on Pituitary Gonadotrophs

PACAP directly affects the pituitary gland via the portal circulation. In addition, PACAP is expressed in gonadotrophs as well as folliculostellate cells of the adenohypophysis [46,47], suggesting that PACAP may serve as both a classical releasing factor and as an autocrine-paracrine factor within the pituitary. PACAP itself can stimulate gonadotropin subunit gene expression [48]. In addition, similarly to GnRH, pulsatile stimulation with PACAP specifically induces gonadotropin subunits in gonadotrophs, in which high-frequency PACAP pulses increase LH expression to a larger extent than that induced by low-frequency PACAP pulses. In contrast, lower frequencies of PACAP pulses preferentially increase FSHb-subunit expression [49]. 

### 4.3. Interaction between GnRHR and PAC1R in Pituitary Gonadotrophs

**Figure 2:**
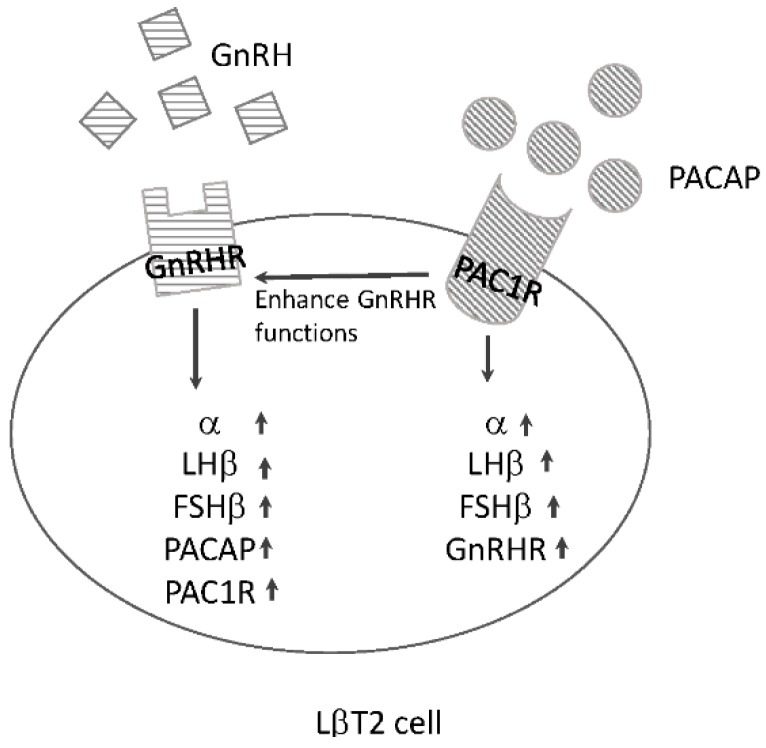
Schematic summary of the action of GnRH and PACAP in pituitary gonadotrophic LβT2 cells. In static cultures of LβT2 cells, both GnRH and PACAP increase the expression of gonadotropin α-, LHβ- and FSHβ-subunits. GnRH stimulates the expression of PACAP and its receptor PAC1R, while GnRH receptor expression is stimulated by PACAP. The presence of PAC1R enhances GnRH’s effects on gonadotropin expression.

Both GnRH and PACAP have the ability to stimulate gonadotropin subunit expression, but the signals of these two neuropeptides interact in pituitary gonadotrophs. GnRH can stimulate PACAP expression in the primary cultures of pituitary gonadotrophs as well as in the gonadotrophic cell line LbT2 [50,51]. GnRH also increases PAC1R expression [52]. Conversely, the expression levels of GnRHR are increased by PACAP stimulation [49]. Gonadotropin subunit expression is also influenced by the expression levels of GnRHR and PAC1R in gonadotrophs. Expression of the LHb-subunit is optimally stimulated at a relatively high cell surface density of GnRHR, whereas FSHb gene expression is favored at a low density of GnRHR [53]. Increasing amounts of PAC1R in the cells potentiate the effects of PACAP on both LHb- and FSHb-subunit gene expression [51]. Furthermore, increasing the density of cell surface PAC1R potentiates the effect of GnRH on both gonadotropin subunits [54] (Figure 2).

As for receptor signalling, it has been reported that GnRH and PACAP both increase cAMP levels individually, but GnRH prevents PACAP-stimulated cAMP accumulation [55,56]. GnRH-induced activation of the ERK signaling pathway as well as cAMP/PKA pathways are potentiated in the presence of PAC1R [54].

### 4.4. Potential Roles of PACAP and PAC1R in GnRH Pulse Frequency-Dependent Gonadotropin Subunit Expression

**Figure 3:**
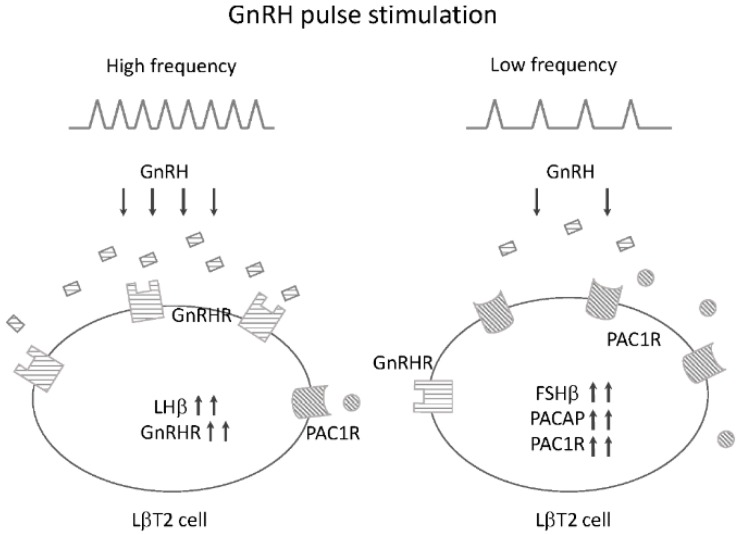
Schematic summary of the changes in pituitary gonadotrophic LβT2 cells by high- and low-frequency pulsatile GnRH stimulation. High-frequency GnRH pulse stimulation preferentially stimulates LHβ-subunit and GnRH receptor expression. On the other hand, FSHβ-subunit, PACAP, and PAC1R gene expression predominantly occurs under low-frequency GnRH pulses.

As described above, GnRH and PACAP signals interact and modulate their effects on gonadotrophs by changing their receptor levels. In pulsatile GnRH stimulation, GnRHRs within the gonadotroph are upregulated at higher frequencies of GnRH pulses [57]. As for PACAP and its receptor, when cells were exposed to lower frequencies of GnRH pulses, expression of PACAP and PAC1R was predominantly increased compared to when cells were stimulated with high frequencies of GnRH pulses [52], suggesting that gonadotrophs are functionally altered to produce more FSHb-subunit by changing PACAP and PAC1R expression (Figure 3). Indeed, we have observed that an increase in FSHb-subunit gene expression by GnRH was significantly prevented in the presence of a PAC1R antagonist [52], suggesting the possibility that PACAP and PAC1R produced within gonadotrophs may modulate GnRH pulse frequency-dependent gonadotropin regulation in an autocrine/paracrine manner. 

## 5. Prolactin Synthesis and Secretion from Pituitary Lactotrophs

Prolactin, which is released from lactotrophs in the anterior pituitary, has a great diversity of actions. In female reproductive tissues, it plays a dominant role in the breast including development and growth of mammary glands and synthesis and secretion of milk [58]. Prolactin is necessary to achieve pregnancy [59] and maintain normal reproductive cycles [60]. Yet, excess prolactin has a number of adverse effects on various steps in the reproductive axis [61]. Although previous studies firmly established that dopamine is the predominant physiological factor inhibiting prolactin release from pituitary lactotrophs [8], prolactin synthesis and release are also regulated by prolactin-releasing factors.

### 5.1. Thyrotropin-Releasing Hormone (TRH) as a Prolactin-Releasing Factor

After its initial isolation and characterization, TRH was demonstrated to cause a rapid release of prolactin from rat pituitary cell cultures [62]. In humans, intravenous injection of TRH also induces the release of prolactin [63]. However, it remains unknown whether TRH has a physiological role in the regulation of prolactin. 

TRH stimulates inositol phospholipid metabolism by activating the TRH receptor in lactotrophs. Subsequently, it stimulates PKC-related pathways and calcium release from intracellular calcium storage sites [64]. ERK is activated by TRH via PKC-dependent and PKC-independent pathways [65], and activated ERK is inactivated by dual-specificity threonine/tyrosine MAPK phosphatase [66,67]. We have previously demonstrated the importance of ERK signaling activation in TRH-induced prolactin gene expression [68,69]. 

### 5.2. Effect of PACAP on Prolactin-Producing Cells

PACAP was initially believed to be devoid of prolactin-releasing activity when it was first isolated [10]. Similarly, in ovine and bovine pituitary cells, PACAP had no effect on prolactin release [70,71]. Conversely, other studies demonstrated the stimulating effect of PACAP on prolactin release [72,73]. In prolactin-producing model GH3 cells that originate from rat pituitary somatolactotrophs, PACAP increases the synthesis and secretion of prolactin with a concomitant activation of ERK and cAMP/PKA pathways [74].

### 5.3. Interaction between the TRH Receptor and PAC1R in Somatolactotrophic GH3 Cells

**Figure 4:**
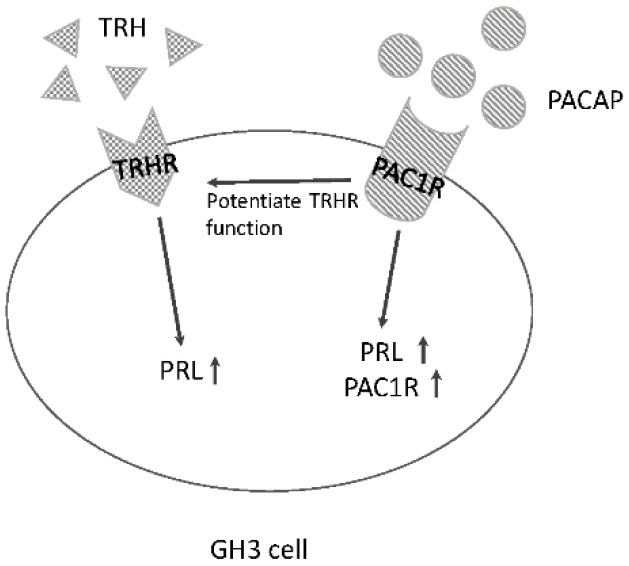
Schematic summary of the action of TRH and PACAP in pituitary prolactin-producing GH3 cells. Both TRH and PACAP increase prolactin gene expression. PACAP also increases the expression of PAC1R. In addition, the presence of PAC1R potentiates the effect of TRH on prolactin gene expression.

GH3 cells respond to PACAP by increasing prolactin gene expression. When PAC1R is overexpressed in these cells, the effect of PACAP on prolactin expression is more clearly observed. In addition, prolactin gene expression induced by the same concentration of PACAP was significantly increased after transfecting GH3 cells with increasing amounts of PAC1R expression vector. These observations suggest that the presence of much more PAC1R within the cells allows them to respond to PACAP and produce more prolactin. Intriguingly, combined stimulation with TRH and PACAP potentiated their individual effects. In addition, increasing PAC1R expression by transfecting increasing amounts of PAC1R expression vectors potentiates the effect of TRH on prolactin expression [75]. This observation supports the hypothesis that the presence of PAC1R itself might augment the ability of TRH to stimulate prolactin synthesis. This phenomenon is quite similar to that observed in pituitary gonadotrophs, in which an increasing density of cell surface PAC1R potentiates the effect of GnRH on both gonadotropin subunits [54]. It was revealed that although TRH does not have the ability to stimulate PAC1R expression, PACAP itself increases PAC1R expression in GH3 cells [75] (Figure 4).

It is a well-known phenomenon observed in pituitary gonadotrophs that continuous exposure to GnRH leads to desensitization of GnRHR and decreases gonadotropin secretion, subsequently preventing sex steroid synthesis [76]. This concept is applied to the medical suppression of ovarian function in patients with sex steroid-dependent diseases. Similarly, it has been reported that prolonged treatment with TRH also decreased the number of TRH receptors in pituitary cells [77]. Interestingly, prolonged stimulation with TRH eliminated the stimulatory effect of TRH on prolactin gene expression, whereas prolonged treatment with PACAP also blunted the effect of PACAP [78], suggesting that TRH and PACAP could desensitize both their own receptors and each other’s receptors in prolactin-producing cells.

## 6. Conclusions

In this review, we summarized the role of PACAP and PAC1R in GnRH-, gonadotropin-, and prolactin-producing neurons. Although all of these hormones are controlled by their principle regulators, PACAP itself can modify the hormone-producing ability of these cells through PAC1R. In addition, considering the observation that increasing amounts of PAC1R in gonadotrophic and lactotrophic cell lines potentiate their hormone-producing ability by their secretagogues, the G protein-coupled PAC1R may affect other receptor functions. In addition to the scenarios described here for PAC1R, complex interactions between different types of receptors may coordinately work in hormone-secreting cells.
